# Stimulation of *Vibrio vulnificus* Pyruvate Kinase in the Presence of Glucose to Cope With H_2_O_2_ Stress Generated by Its Competitors

**DOI:** 10.3389/fmicb.2018.01112

**Published:** 2018-05-29

**Authors:** Hey-Min Kim, Chang-Kyu Yoon, Hyeong-In Ham, Yeong-Jae Seok, Young-Ha Park

**Affiliations:** School of Biological Sciences and Institute of Microbiology, Seoul National University, Seoul, South Korea

**Keywords:** adaptation to H_2_O_2_ stress, bacterial–fungal interaction, competition for glucose, phosphotransferase system, pyruvate kinase

## Abstract

The bacterial phosphoenolpyruvate (PEP):carbohydrate phosphotransferase system (PTS) regulates a variety of cellular processes in addition to catalyzing the coupled transport and phosphorylation of carbohydrates. We recently reported that, in the presence of glucose, HPr of the PTS is dephosphorylated and interacts with pyruvate kinase A (PykA) catalyzing the conversion of PEP to pyruvate in *Vibrio vulnificus*. Here, we show that this interaction enables *V. vulnificus* to survive H_2_O_2_ stress by increasing pyruvate production. A *pykA* deletion mutant was more susceptible to H_2_O_2_ stress than wild-type *V. vulnificus* without any decrease in the expression level of catalase, and this sensitivity was rescued by the addition of pyruvate. The H_2_O_2_ sensitivity difference between wild-type and *pykA* mutant strains becomes more apparent in the presence of glucose. Fungi isolated from the natural habitat of *V. vulnificus* retarded the growth of the *pykA* mutant more severely than the wild-type strain in the presence of glucose by glucose oxidase-dependent generation of H_2_O_2_. These data suggest that *V. vulnificus* has evolved to resist the killing action of its fungal competitors by increasing pyruvate production in the presence of glucose.

## Introduction

Microorganisms usually have complex ecological interactions with many different species in natural environments. To ensure their survival and prosperity in a wide variety of ecological conditions, microbes need to decide whether to cooperate or compete with other species for limited nutritional resources and defend themselves from potential competitors and predators. Therefore, they possess multiple regulatory systems to sense and adapt to the constantly changing environment. One bacterial sensory system that plays an important part in monitoring nutritional states and modulating rapid physiological adjustment to environmental changes is the phosphoenolpyruvate (PEP):carbohydrate phosphotransferase system (PTS) (Deutscher et al., 2006). This system uses PEP as the energy source for the concomitant transport and phosphorylation of its carbohydrate substrates in a process termed group translocation (Kundig et al., 1964; Roseman, 1969).

The PTS is composed of two general proteins, enzyme I (EI) and HPr, which are used for most PTS carbohydrates, and the carbohydrate-specific permeases, commonly referred to as the enzyme II (EII) complexes (Meadow et al., 1990; Postma et al., 1993). In the presence of a PTS carbohydrate, a phosphoryl group is sequentially transferred from PEP to EI, HPr, the EII complex and finally to the incoming carbohydrate. Therefore, phosphorylation of the PTS components increases in the absence and decreases in the presence of a PTS carbohydrate substrate such as glucose. In this way, the PTS carbohydrate transport system monitors nutritional changes in the environment and regulates a variety of metabolic processes through the phosphorylation state-dependent protein–protein interactions of the components involved (Deutscher et al., 2014; Park et al., 2015).

We recently reported that, in the presence of glucose, HPr of the PTS is dephosphorylated and interacts with pyruvate kinase A (PykA), catalyzing the conversion of PEP to pyruvate in *Vibrio vulnificus* (Kim et al., 2015). In *V. vulnificus*, there are two pyruvate kinase isozymes, PykF and PykA, and PykF is the major pyruvate kinase. Only PykA, but not PykF interacted with HPr in *V. vulnificus*. While we could not make a *pykF* deletion mutant even after repeated trials, *pykA* was dispensable under normal growth conditions in *V. vulnificus* (Kim et al., 2015). Interestingly, although PykA and HPr of *Escherichia coli*, belonging to the same γ-proteobacterial group as *V. vulnificus*, share 71 and 76% amino acid sequence identities, respectively, with their orthologs in *V. vulnificus*, the regulatory interaction of HPr with PykA is not observed in *E. coli*. This observation prompted the question of why the two very close species should have developed different regulatory mechanisms for the same enzyme. The primary habitat of *E. coli* is the lower intestine of warm-blooded animals (Whittam, 1989), whereas *V. vulnificus* is usually present in coastal marine environments (Bhadury et al., 2011; Horseman and Surani, 2011). Antagonistic interactions between bacteria and fungi in competing for a common substrate such as glucose have been documented in many habitats including an aquatic environment (Mille-Lindblom et al., 2006; Arvanitis and Mylonakis, 2015). Here, we show that fungi isolated from the natural habitat of *V. vulnificus* retarded the growth of the *pykA* mutant more severely than the wild-type strain in the presence of glucose by glucose oxidase-dependent generation of H_2_O_2_. Interestingly, the HPr-PykA interaction enables *V. vulnificus* to survive H_2_O_2_ stress by increasing pyruvate production in the presence of glucose. These data suggest that *V. vulnificus* has evolved to resist the killing action of its competitors by increasing pyruvate production in the presence of glucose.

## Materials and Methods

### Growth Conditions

*Vibrio vulnificus* strains were cultured in Luria-Bertani medium containing 2.5% NaCl (LBS) or M9 minimal medium containing 0.2% casamino acids and 2.5% NaCl (M9S) at 30°C. All *E. coli* strains were grown in LB medium at 37°C. Fungal strains were cultured in potato dextrose agar (PDA) plates or M9S medium at 30°C. Details of strain and plasmid constructions are provided in the **Table 1**.

**Table 1:** Bacterial and fungal strains and plasmids used in this study.

Strains or plasmids	Genotypes and/or descriptions	Reference
***V. vulnificus* strains**		
CMCP6	Clinical isolate	Kim et al., 2003
CMCP6 Δ*pykA*		Kim et al., 2015
***E. coli* strains**		
MG1655		
MG1655 Δ*pykA*		This study
SM10 λ*pir*	*thi-1 thr leu tonA lacY supE recA*::Rp4-2-Tc::Mu λ*pir*; Km^r^	Simon et al., 1983; Amadi, 2016
**Fungal strains**		
*Aspergillus fumigatus*	SFC201500303-M02 (isolated from seaweed and mudflat in marine environments)	Lee et al., 2016
*Aspergillus welwitschiae*	SFC20160317-M21 (isolated from seaweed and mudflat in marine environments)	Lee et al., 2016
**Plasmids**		
pRK415	Broad host range vector, IncP *ori*, *oriT* of RK2; Tc^r^	Keen et al., 1988
pRK-H15A	His15 of vHPr in pRK-vHPr was mutated to Ala	Kim et al., 2015
pRK-PKA	*V. vulnificus pykA* promoter and ORF was cloned into BamHI/PstI sites of pRK415	Kim et al., 2015
pRK-PKA&H15A	*V. vulnificus ptsH* promoter and vHPr(His15Ala) was cloned into PstI/HindIII sites of pRK-PKA	Kim et al., 2015
pRK-vePKA	*V. vulnificus pykA* promoter and chimeric ORF was cloned into PstI/HindIII sites of pRK415	This study
pRK-vePKA&H15A	*V. vulnificus ptsH* promoter and vHPr(His15Ala) was cloned into BamHI/PstI sites of pRK-vePKA	This study

### Induction of VBNC State

*Vibrio vulnificus* cells in late log phase were harvested by centrifugation at 10,000 × *g* for 10 min and washed twice with artificial sea water [ASW; 60 mM NaCl, 20 mM MgSO_4_⋅7H_2_O, 20 mM KCl, 2 mM CaCl_2_⋅2H_2_O, 50 mM Tris-HCl (pH 8.0)]. Cells were diluted with ASW to a cell density of 10^8^ CFU/ml, transferred to sterile microcentrifuge tubes, and incubated at 4°C in the dark without shaking. Then the total cell number, viability, and culturability were assessed from each tube 0, 2, 4, and 11 days after incubation. Total and viable cell numbers were determined using a Live/Dead BacLight Bacterial Viability Kit (Life Technologies) according to the manufacturer’s instructions. Samples stained with SYTO 9 dye and propidium iodide were spotted on a 1% agarose pad made with Phosphate Buffered Saline (PBS) on a glass slide. Cells were visualized using a Deltavision Restoration Microscope System (GE Healthcare Life Sciences). The numbers of bacterial cells were then counted and calibrated to give total and viable counts per milliliter. To assess the culturability of *V. vulnificus* cells, samples were spread on LBS plates in triplicate, and incubated at 30°C for 48 h before the CFU numbers were assessed.

### Determination of Bacterial Survival Under H_2_O_2_ Stress Condition

*Vibrio vulnificus* cells were inoculated into the appropriate liquid media containing different combinations of H_2_O_2_ and pyruvate at a density of approximately 10^5^ cells/ml. Optical density of cultures was measured at 600 nm with a spectrophotometer or a 96-well microplate reader (TECAN Spark^TM^ 10 M multimode microplate reader, Männedorf, Switzerland). To obtain culture filtrates of fungi, *Aspergillus fumigatus* or *A. welwitschiae* was cultured overnight in M9S medium containing glucose or galactose and the culture media were collected and filtrated through a 0.25 μm pore size membrane (Millipore Corp.). Growth on solid medium was assessed by serially (fourfold) diluting cells and spotting 2 μl onto the TCBS (Thiosulfate-citrate-bile salts-sucrose) agar plates containing different combinations of H_2_O_2_ and pyruvate, followed by growth for 24 h.

### Detection of Catalase by Native Gel Electrophoresis

*Vibrio vulnificus* strains were cultured in LBS medium with or without 0.8 mM H_2_O_2_. At the exponential growth phase (OD_600_ ∼ 0.5), cells were collected by centrifugation and the cell pellet washed with 50 mM potassium phosphate buffer (pH 7.0). Cells were then disrupted by passing twice through a French pressure cell at 10,000 psi and centrifuged at 5,000 × *g* for 20 min at 4°C. The amount of protein in a cell lysate was determined by the Bradford assay by using bovine serum albumin as the standard. After separation on a 10% non-denaturing poly-acrylamide gel, the locations of catalase were visualized by staining the gel with a solution containing 2% K_3_Fe(CN)_6_ and 2% FeCl_3_ (Wayne and Diaz, 1986).

### Determination of Glucose Oxidase Activities

*Aspergillus* strains were cultured in M9S media containing 0.2% glucose for 2 days. The fungal cells were then harvested by filtration and the cell pellet was washed twice with sodium acetate buffer (pH 5.5). Cells were then disrupted by sonication and passed twice through a French pressure cell at 10,000 psi, and centrifuged at 5,000 × *g* for 20 min at 4°C. The amount of protein in a cell lysate was determined by the Bradford assay by using bovine serum albumin as the standard. For in-gel glucose oxidase assays, proteins in the cell extracts were separated on a 16.5% non-denaturing poly-acrylamide gel containing entrapped peroxidase [5 ml separating gel solution containing 19.2 purpurogallin units of horseradish peroxidase (HRP, Sigma-Aldrich)], and the locations of glucose oxidase were visualized by staining the gel with a solution containing 30 mM glucose and 17 mM *ortho*-phenylenediamine (Mateescu et al., 2012). We also determined glucose oxidase activities from fungal cell extracts at 30°C spectrophotometrically by monitoring the change in *A*_460_ due to oxidation of *o*-dianisidine by HRP, using 8.3 as the molar extinction coefficient (Kelley and Reddy, 1986). The reaction mixture consisted of sodium acetate buffer (50 mM, pH 5.5), 50 μM *o*-dianisidine, 1.6% glucose, 6 units of HRP, and 250 μl of crude extracts in a total volume of 1 ml. The purified glucose oxidase of *A. niger* (Sigma-Aldrich) was used as a positive control. The reaction mixture was bubbled with oxygen for 5 min before addition of glucose oxidase.

### Detection of H_2_O_2_ Level

The H_2_O_2_ levels were determined using the hydrogen peroxide assay kit (BioVision), according to the manufacturer’s instructions. Briefly, control media or culture filtrates of fungal strains were mixed with a 50 μl reaction mixture containing assay buffer, OxiRed probe solution, and HRP solution, and incubated at room temperature for 10 min. Concentrations of H_2_O_2_ were determined with a microplate reader at 570 nm.

## Results

### *Vibrio vulnificus* PykA Activity Confers Resistance to H_2_O_2_

**FIGURE 1:**
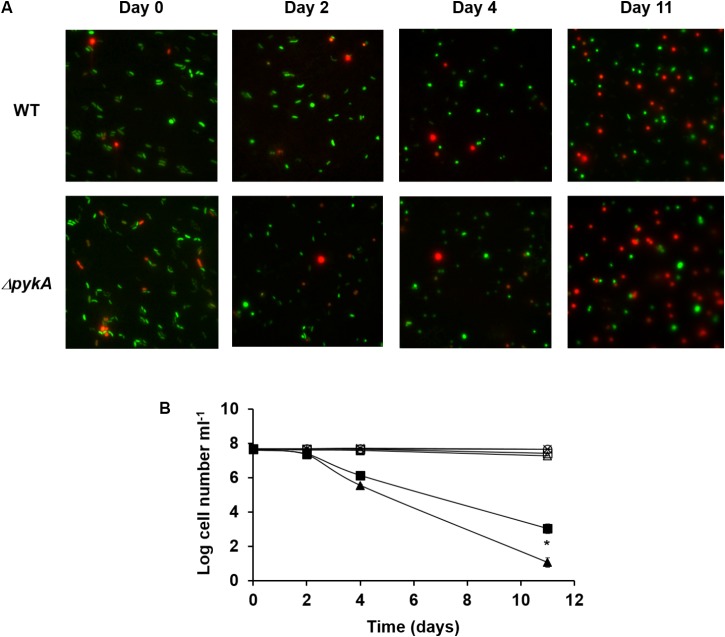
Induction of viable but non-culturable (VBNC) state by *Vibrio vulnificus* in the artificial sea water on incubation at 4°C. **(A)** Microscopic pictures of wild-type *V. vulnificus* CMCP6 and *pykA* mutant strains after 0, 2, 4, and 11 days incubation at 4°C. Samples were stained with the Live/Dead BacLight Bacterial Viability Kit (Life Technologies). The two color fluorescence assay uses green nucleic acid stain that penetrates both live and dead cells, while the red propidium iodide stain is excluded from live cells. Representative data from three independent experiments are shown. **(B)** Total cell counts, viable cell counts, and plate counts were determined. Plate counts are given in cfu ml^-1^. ×, total cell counts of the wild-type *V. vulnificus* CMCP6 culture; ○, total cell counts of the *pykA* mutant culture; □, viable cell counts of the wild-type *V. vulnificus* CMCP6 culture; Δ, viable cell counts of the *pykA* mutant culture; ■, plate counts of the wild-type *V. vulnificus* CMCP6 culture; ▲, plate counts of the *pykA* mutant culture. The results are presented as the mean ± standard deviations of three independent measurements. Statistical significance for the VBNC cells on Day 11 was determined by Student’s *t*-test (^∗^*P* < 0.05, *n* = 3).

It is known that, like many other Gram-negative bacteria, *V. vulnificus* is induced into a viable but non-culturable (VBNC) state by incubation at low temperatures (Oliver, 2010). Several studies have provided evidence for the involvement of reactive oxygen species (ROS) in the VBNC state of *V. vulnificus* by showing that a significant portion of the VBNC population of *V. vulnificus* can be resuscitated if a H_2_O_2_-scavenging agent such as catalase or pyruvate is introduced to the culture medium (Bogosian et al., 2000; Kong et al., 2004). While catalase decomposes H_2_O_2_ into oxygen and water through its enzymatic activity (Loew, 1900), pyruvate reacts non-enzymatically with H_2_O_2_ to yield carbon dioxide, water, and acetic acid (Bunton, 1949). Because pyruvate is a product of the reaction catalyzed by pyruvate kinases, we assumed that the PykA activity could be related to an adaptive defense against H_2_O_2_ stress in *V. vulnificus*. To test whether there were any differences in the development of the VBNC state between the wild-type and *pykA* mutant strain, we determined the viability and culturability of each strain at low temperature (**Figure 1**). The VBNC state was induced in the artificial sea water by incubation at 4°C. As expected, the *pykA* mutant strain lost the ability to form colonies at a somewhat faster rate than the wild-type strain (**Figure 1B**). These results suggested that the development of the VBNC state of *V. vulnificus* could be influenced by the activity of PykA.

**FIGURE 2:**
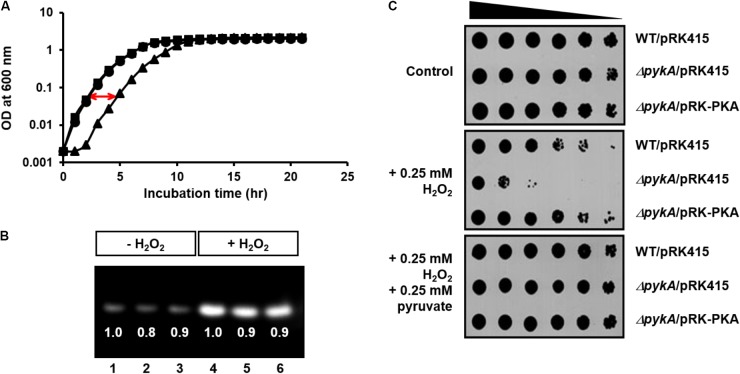
Effects of PykA activity and pyruvate on resistance of *V. vulnificus* to H_2_O_2_ stress. **(A)** Growth curves of wild-type CMCP6 (squares), *pykA* mutant (triangles) and complemented (*pykA*/pRK-PKA, circles) strains in LB supplemented with 2.5% NaCl (LBS medium) in the presence of 0.8 mM H_2_O_2_. **(B)** The relative expression levels of catalase in wild-type CMCP6 (lanes 1 and 4), *pykA* mutant (lanes 2 and 5) and the complemented strains (lanes 3 and 6) in LBS medium in the presence and absence of 0.8 mM H_2_O_2_. Same amounts of proteins in cell lysates were loaded in each lane. **(C)** Stationary-phase cells of the indicated *V. vulnificus* strains were serially diluted fourfold from 0.25 × 10^8^ cells/ml and 2-μl aliquots were spotted onto LBS medium containing different combinations of H_2_O_2_ and sodium pyruvate as indicated. Representative data from at least three independent experiments are presented.

To study whether the effect of PykA on the VBNC state is related to the synthesis of pyruvate, a H_2_O_2_-scavenging agent, we determined the growth curves of wild-type and *pykA* mutant strain in the presence of 0.8 mM H_2_O_2_. While the two strains did not show a significant difference in growth in LB supplemented with 2.5% NaCl (LBS medium) (Supplementary Figure S1), the *pykA* mutant exhibited a significantly retarded growth compared to the wild-type in the presence of 0.8 mM H_2_O_2_ (**Figure 2A**). The episomal expression of PykA from pRK-PKA could rescue the H_2_O_2_ sensitivity of the *pykA* mutant, indicating that the increased sensitivity of the mutant to H_2_O_2_ was a direct reflection of the loss of PykA.

The other H_2_O_2_ scavenging agent, catalase, is a common enzyme found in nearly all living organisms exposed to oxygen and protects cells from oxidative damage (Loew, 1900). This raises the possibility that the decreased H_2_O_2_ resistance of the *pykA* mutant could be due to a decreased catalase expression or activity in *V. vulnificus* cells. Therefore, we compared the relative catalase activities between the wild type, the *pykA* mutant, and a complemented strain (*pykA*/pRK-PKA) in the presence and absence of H_2_O_2_ (**Figure 2B**). While the addition of H_2_O_2_ to the culture medium resulted in a significant induction of catalase in all strains tested, no significant difference could be detected in the catalase expression levels and activities among the three strains (**Figure 2B**).

To confirm the effects of PykA activity and pyruvate on resistance to H_2_O_2_ stress, the H_2_O_2_ sensitivity assays were performed on LBS medium containing different combinations of H_2_O_2_ and pyruvate (**Figure 2C**). In agreement with observations in **Figure 2A**, the *pykA* mutant exhibited a significantly decreased survival on the same medium but supplemented with 0.25 mM H_2_O_2_. Intriguingly, the addition of exogenous pyruvate fully protected the cells from H_2_O_2_ killing. On the contrary, there was no significant difference in H_2_O_2_ sensitivity between the wild-type *E. coli* MG1655 and its otherwise isogenic *pykA* mutant strain (Supplementary Figure S2). These results indicate that the role of PykA in H_2_O_2_ resistance is not conserved among bacteria: PykA-dependent production of pyruvate seems to play an important role in H_2_O_2_ scavenging in *V. vulnificus*, whereas catalase is known to be the primary scavenger in *E. coli* when H_2_O_2_ levels are high (Seaver and Imlay, 2001a,b).

### HPr Increases H_2_O_2_ Resistance by Stimulating PykA Activity in the Presence of Glucose

**FIGURE 3:**
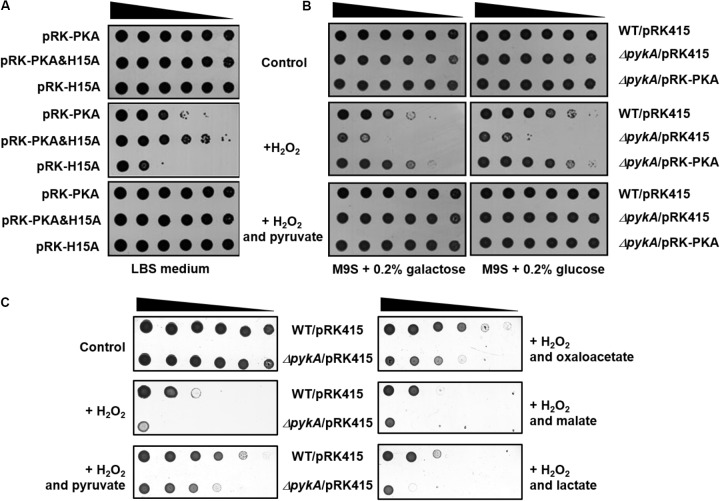
Effects of PykA and dephospho-HPr on resistance to H_2_O_2_ stress. **(A)** The *pykA* mutant cells harboring the indicated plasmids were serially diluted fourfold from 10^8^ cells/ml, and 2-μl aliquots were spotted onto LBS medium containing different combinations of H_2_O_2_ and sodium pyruvate as indicated. pRK-PKA expresses *V. vulnificus* PykA from its own promoter and pRK-PKA&H15A expresses both PykA and HPr(H15A) (a phosphorylation site mutant mimicking the dephosphorylated form) from their own promoters. **(B)** The indicated strains were serially diluted fourfold from 10^8^ cells/ml, and 2-μl aliquots were spotted onto M9S medium containing a carbohydrate, H_2_O_2_ and sodium pyruvate either alone or in different combinations as indicated. It should be noted that M9S agar plates were supplemented with H_2_O_2_ at half the concentration added to LBS plates because cells were more sensitive to H_2_O_2_ in M9S plates than in LBS plates. **(C)** The indicated strains were serially diluted fourfold from 10^8^ cells/ml, and 2-μl aliquots were spotted onto LBS medium containing 0.25 mM H_2_O_2_ and 0.25 mM indicated supplements. Representative data from at least three independent experiments are presented.

We have recently shown that the presence of glucose in culture medium increases the dephosphorylated form of HPr, which tightly interacts with PykA, stimulating its activity by decreasing the Km for PEP in *V. vulnificus* (Kim et al., 2015). To examine whether the stimulation of pyruvate production by dephosphorylated HPr affects H_2_O_2_ resistance *in vivo*, we constructed an expression vector for a non-phosphorylatable (His15 to Ala) mutant of *V. vulnificus* HPr (pRK-H15A) and a vector coexpressing PykA and HPr(H15A) of *V. vulnificus* (pRK-PKA&H15A in **Table 1**). As shown in **Figure 3A**, the *pykA* mutant strain carrying pRK-PKA was more resistant to H_2_O_2_ than the mutant carrying pRK-H15A, whereas transformation of the mutant with pRK-PKA&H15A further increased H_2_O_2_ resistance compared with the strain carrying pRK-PKA, indicating that HPr(H15A) confers increased resistance to H_2_O_2_ stress in the presence of PykA. It should be noted that the expression of HPr(H15A) alone had little effect on H_2_O_2_ resistance of the *pykA* mutant (Supplementary Figure S3), suggesting that the effect of HPr(H15A) on H_2_O_2_ resistance is mediated by the stimulation of PykA.

The interaction with and activation of PykA by HPr is observed in *V. vulnificus* but not in *E. coli* and this species specificity of the HPr-PykA interaction is determined by the C-terminal domain of PykA (Kim et al., 2015). To examine whether H_2_O_2_ resistance of the *pykA* mutant harboring pRK-PKA&H15A was due to the specific interaction of HPr(H15A) with PykA, we constructed an expression vector for vePykA (pRK-vePKA) in which the C-terminal domain (amino acids 334 to 480) was replaced with that of *E. coli* PykA and a vector coexpressing vePykA and HPr(H15A) (pRK-vePKA&H15A). The *pykA* mutant transformed with pRK-vePKA was more resistant to H_2_O_2_ than the mutant carrying pRK415 or pRK-H15A (Supplementary Figures S3, S4). However, the mutant carrying pRK-vePKA&H15A exhibited little difference in H_2_O_2_ resistance compared with the strain carrying pRK-vePKA (compare **Figure 3A** and Supplementary Figure S4), implying that the interaction and stimulation of PykA by dephospho-HPr is important for the increased H_2_O_2_ resistance of *V. vulnificus*.

It is known that HPr is mostly dephosphorylated in the medium supplemented with glucose, whereas it is predominantly in the phosphorylated state in the medium supplemented with galactose in *V. vulnificus* (Kim et al., 2015). The effect of the carbohydrate type on H_2_O_2_ sensitivity was investigated by spotting serially diluted cells onto agar plates with M9 medium supplemented with 0.2% casamino acids and 2.5% NaCl (M9S medium), containing different combinations of carbohydrate, H_2_O_2_, and pyruvate (**Figure 3B**). The wild-type strain and the *pykA* mutant strain harboring the pRK-PKA plasmid were considerably less sensitive to H_2_O_2_ in the presence of glucose than in the presence of galactose, whereas no effect of the carbohydrate type was seen in the *pykA* mutant. Because the catalase expression level was little affected by the presence of these carbohydrates (Supplementary Figure S5), this carbohydrate effect is likely to be mediated solely through the phosphorylation state-dependent stimulation of PykA activity by HPr. More specifically, dephosphorylated HPr interacts with and stimulates PykA so that an increase in PykA-mediated pyruvate production confers resistance to H_2_O_2_ stress in the presence of glucose.

Since the first description of the chemical reaction between α-ketoacids and H_2_O_2_ producing acetate and CO_2_ in 1904 (Holleman, 1904), many studies have confirmed this non-enzymatic H_2_O_2_-scavenging reaction (Bunton, 1949; Desagher et al., 1997; Troxell et al., 2014; Lopalco et al., 2016). Because α-ketoacids such as pyruvate and oxaloacetate are known to play vital roles in central carbon metabolism as well as to serve as H_2_O_2_ scavengers, it was necessary to determine whether the protective effect of pyruvate against H_2_O_2_ resulted from its ability to react with H_2_O_2_ or from its requisite role in central metabolism. To verify this, we tested the H_2_O_2_ sensitivity of the *pykA* mutant by spotting on LBS medium containing H_2_O_2_ and an α-ketoacid (pyruvate or oxaloacetate) or an α-hydroxyacid (lactate or malate) (**Figure 3C**). *V. vulnificus* has both lactate dehydrogenase and malate dehydrogenase, which catalyze the reversible oxidation of lactate and malate to pyruvate and oxaloacetate, respectively, coupled to the reduction of NAD^+^ to NADH. Therefore, if the protective effect of pyruvate is due to an increased metabolic flux, the same concentration of lactate and malate should exert a higher, or at least similar, protective effect against H_2_O_2_ than pyruvate and oxaloacetate, respectively. As shown in **Figure 3C**, However, only α-ketoacids, pyruvate, and oxaloacetate, could rescue the H_2_O_2_ sensitivity of the *pykA* mutant. Furthermore, the addition of citrate to LBS medium did not rescue the *pykA* mutant cells from H_2_O_2_ stress (Supplementary Figure S6). These data suggest that the protective effect of pyruvate is not due to an increased metabolic flux.

Bacterial mutants deleted for a glycolytic gene usually show a severe growth defect in LB and minimal medium due to limited metabolic capabilities (Irani and Maitra, 1977; Commichau et al., 2013). However, there was no difference in growth between the wild type and the *pykA* mutant strain in LBS (Supplementary Figure S1). These results suggest that the *V. vulnificus pykA* mutant does not have any significant metabolic perturbation. We have previously shown that the intracellular pyruvate concentration drastically increased after the addition of glucose in the wild-type strain. Notably, the intracellular pyruvate concentration also increased in *pykA* mutant cells after the addition of glucose, but to a lesser extent. In the presence of galactose, the pyruvate concentration was similar or slightly higher in wild-type cells compared to the *pykA* mutant (Kim et al., 2015). Furthermore, there was no significant difference in the acetate concentrations between the two strains in LBS medium and M9S medium supplemented with glucose (Supplementary Figure S7). These data also suggest that the phenotype of the *pykA* mutant is not due to the limited metabolic flux.

### PykA Confers Resistance to H_2_O_2_ Stress Caused by Fungal Neighbors

**FIGURE 4:**
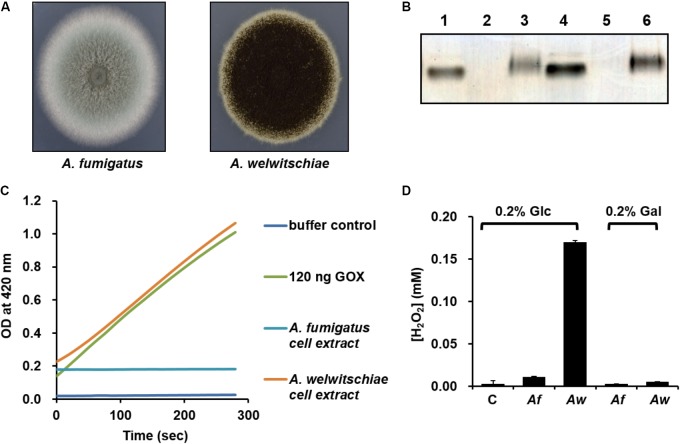
Assay of glucose oxidase from *Aspergillus* strains and analysis of H_2_O_2_ concentrations from *Aspergillus* culture supernatants. **(A)** Colonies of *Aspergillus fumigatus* and *A. welwitschiae* isolated from the natural habitat of the *V. vulnificus* CMCP6 strain. **(B)** In-gel glucose oxidase assay on a 16.5% poly-acrylamide gel containing entrapped peroxidase. Lanes 1 and 4, Type X-S *A. niger* glucose oxidase (Sigma-Aldrich) as control (25 and 50 ng, respectively); lanes 2 and 5, *A. fumigatus* crude extract (15 and 30 μg proteins, respectively); lanes 3 and 6, *A. welwitschiae* crude extract (15 and 30 μg proteins, respectively). **(C)** Glucose oxidase (GOX) activity was determined by a coupled enzyme assay, in which GOX oxidizes glucose resulting in the production of H_2_O_2_ that reacts with *o*-dianisidine in the presence of peroxidase to form a colored product. The intensity of the brown color was measured at 460 nm. **(D)** The H_2_O_2_ levels in M9S media containing glucose or galactose after overnight culture of *A. fumigatus* (*Af*) and *A. welwitschiae* (*Aw*). C, M9S medium without fungi. The results are presented as the mean ± standard deviations (*n* = 3).

Although the *pykA* mutant showed a higher rate of VBNC cell formation compared to the wild-type strain, this phenotype does not seem to be dependent on the presence of glucose, since *V. vulnificus* enters the VBNC state in response to a temperature downshift and cells in the VBNC state have very low metabolic activity (Ayrapetyan et al., 2015). Therefore, we assumed that PykA may play other glucose-dependent role(s) in the natural habitat of *V. vulnificus*. Since the regulatory interaction of HPr with PykA in the presence of glucose was not observed in *E. coli*, we questioned why *V. vulnificus*, but not *E. coli*, cells need a higher PykA activity in the presence of glucose. The primary habitat of *E. coli* is the lower intestine of warm-blooded animals (Whittam, 1989), whereas *V. vulnificus* is usually present in coastal marine environments (Bhadury et al., 2011; Horseman and Surani, 2011). Thus, we assumed that the biochemical and physiological differences between the two species may have arisen from their ecological differences. Recent studies have demonstrated the coexistence of several fungal species such as *Aspergillus*, *Penicillium*, *Fusarium*, and *Rhodotorula* with many bacteria including *V. vulnificus* in the gut of oysters and other shellfishes (Borzykh and Zvereva, 2012; Odu et al., 2012; Froelich and Oliver, 2013; Amadi, 2016; Chen et al., 2016). We could also confirm the coexistence of fungi and *Vibrio* species in the digestive gland of oysters (Supplementary Figure S8). High concentrations of glycogen in the stomach, digestive gland, and other organs of most bivalves including oysters indicate that bivalves may be a good source of glucose for both bacteria and fungi (de Zwaan, 1983; Tikunov et al., 2010). Antagonistic interactions between bacteria and fungi in competing for a common substrate have been documented in many habitats including an aquatic environment (Mille-Lindblom et al., 2006; Arvanitis and Mylonakis, 2015). Interestingly, many fungi have been shown to naturally produce glucose oxidase (GOX), which displays antibacterial activity through the production of H_2_O_2_ in the presence of glucose (Wong et al., 2008). Therefore, we sought to determine whether the glucose-dependent activation of PykA in *V. vulnificus* has anything to do with the glucose-dependent H_2_O_2_ production by its fungal neighbors. Because the *V. vulnificus* CMCP6 strain was originally isolated from a patient in the west coast of Korea (Kim et al., 2003), we tested the GOX activity from the two fungal strains belonging to the *Aspergillus* genus isolated from the west coast of Korea (Lee et al., 2016) (**Figure 4A**). Both zymographic analyses (**Figure 4B**) and coupled enzyme assays with peroxidase and *o*-dianisidine (**Figure 4C**) revealed that *A. welwitschiae* produced a significant amount of GOX when grown in the presence of glucose, whereas little GOX was detected in the crude extract of *A. fumigatus* cells. In accordance with this observation, little H_2_O_2_ was accumulated in the culture medium of *A. fumigatus* regardless of the carbohydrate source, whereas a measurable amount of H_2_O_2_ was accumulated in the culture supernatant of *A. welwitschiae* grown in M9S medium containing glucose, but not in the presence of galactose (**Figure 4D**). These data suggest that some fungal neighbors of *V. vulnificus* can produce a significant amount of H_2_O_2_ through the GOX activity to outcompete surrounding bacterial cells when glucose is available.

**FIGURE 5:**
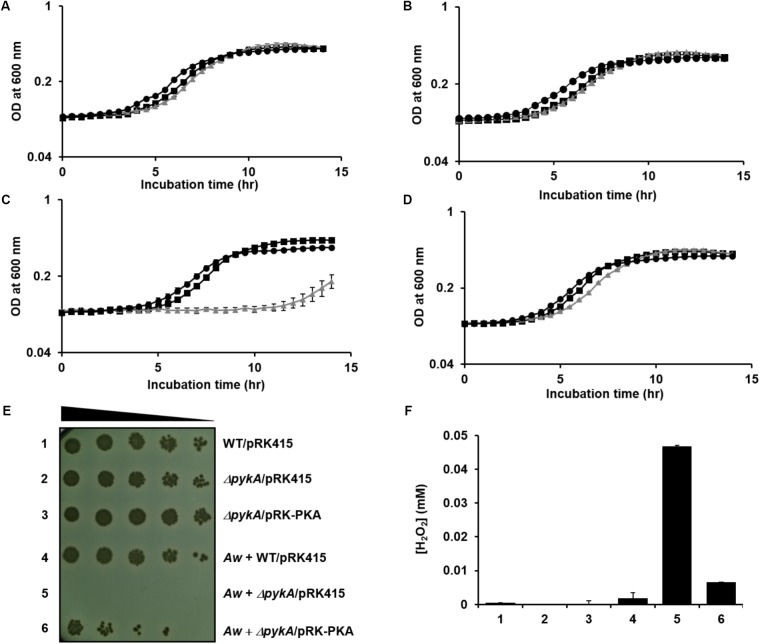
Effect of PykA activity on the survival of *V. vulnificus* against H_2_O_2_ stress generated by its fungal neighbors in the presence of glucose. **(A–D)** Wild-type *V. vulnificus* CMCP6 (squares), the *pykA* mutant (triangles) and the complemented strain (circles) were inoculated in **(A)** fresh M9S medium containing 0.2% glucose, **(B)** the cell-free culture filtrate of *A. fumigatus* grown in M9S medium containing 0.2% glucose, **(C)** the cell-free culture filtrate of *A. welwitschiae* grown in M9S medium containing 0.2% glucose, or **(D)** the cell-free culture filtrate of *A. welwitschiae* grown in M9S medium containing 0.2% glucose and 1 mM sodium pyruvate. Bacterial growths were then monitored by measuring the optical density at 600 nm and presented as the mean ± standard deviations (*n* = 3). **(E)** The indicated strains of *V. vulnificus* were cultured alone (1–3) or with *A. welwitschiae* (*Aw*, 4–6) in M9S medium containing 0.2% glucose. The overnight cultures were serially diluted fourfold and 1.5-μl aliquots were spotted onto TCBS (thiosulfate-citrate-bile salts-sucrose) medium containing 2 μg/ml tetracycline. **(F)** The H_2_O_2_ levels in culture media from **(E)** were determined using the hydrogen peroxide assay kit (BioVision). The results are presented as the mean ± standard deviations (*n* = 3).

To test whether or not a fungus can indeed kill neighboring *V. vulnificus* cells by generating H_2_O_2_ in the presence of glucose, *A. fumigatus* and *A. welwitschiae* were inoculated to M9S medium supplemented with glucose. After overnight incubation, the culture medium was cleared from the fungal cells and mycelia, and then inoculated with wild-type, *pykA* mutant, and the *pykA* mutant carrying pRK-PKA. As expected from H_2_O_2_ sensitivity analyses above, the three strains did not exhibit any growth defects in fresh M9S/glucose medium (**Figure 5A**) and in the cell-free culture filtrate of the *A. fumigatus* in M9S/glucose medium (**Figure 5B**). However, the *pykA* mutant displayed severe growth retardation when inoculated in the filtrate of the *A. welwitschiae* culture in M9S/glucose medium (**Figure 5C**), but this growth retardation was not observed in the cell-free filtrate of the *A. welwitschiae* culture in M9S/glucose medium supplemented with pyruvate (**Figure 5D**). The filtrate of the *A. welwitschiae* culture in M9S/galactose medium did not inhibit growth of the three strains (Supplementary Figure S9). Together, these data indicate that a fungus expressing an active form of GOX can kill *V. vulnificus* cells by generating H_2_O_2_ in the presence of glucose and PykA plays an important role in the protection of *V. vulnificus* cells from H_2_O_2_ stress.

To further confirm the role of PykA in response to H_2_O_2_ stress caused by fungal competitors, the wild-type, *pykA* mutant, and the complemented strain were co-inoculated with *A. welwitschiae* in M9S/glucose medium. After co-culture of *V. vulnificus* strains with the fungus for 24 h, the cultures were serially diluted and then spotted onto selective medium for *V. vulnificus* (**Figure 5E**). When co-cultured with *A. welwitschiae* in M9S/glucose medium, the *pykA* mutant barely grew, whereas obvious growth was detected in the other two strains. Intriguingly, the H_2_O_2_ level in the medium of the co-culture of the fungi and the *pykA* mutant was significantly higher than those from the co-cultures of the fungi and the other two strains (**Figure 5F**). Based on these data, we assumed that *V. vulnificus* resists the H_2_O_2_-mediated killing activity of its fungal competitors by increasing PykA-mediated pyruvate production in the presence of glucose.

## Discussion

We report here a new mechanism that *V. vulnificus* uses to resist the H_2_O_2_-mediated killing of its fungal neighbors. Our earlier work established that dephosphorylated HPr interacts with and stimulates the activity of PykA in the presence of glucose in *V. vulnificus* but this regulatory interaction does not occur in *E. coli* (Kim et al., 2015), even though both species belong to γ-proteobacteria. While the primary habitat of *E. coli* is the lower intestine of warm-blooded animals (Whittam, 1989), the estuarine environment is the primary habitat of *V. vulnificus*. Therefore, we assumed that regulatory functions of the PTS in bacteria might have evolved as adaptations to environmental conditions.

**FIGURE 6:**
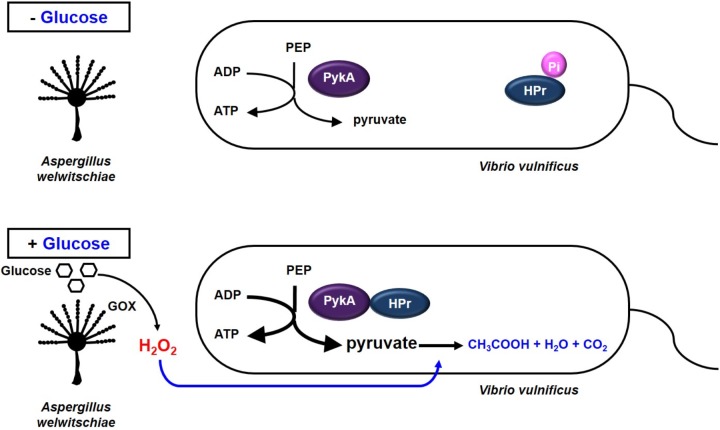
Model showing how *V. vulnificus* cells cope with H_2_O_2_ stress in the presence of glucose. Some fungi are known to inhibit bacterial growth in the presence of glucose by generating H_2_O_2_ through the reaction catalyzed by glucose oxidase. Meanwhile, HPr of the PEP-dependent carbohydrate-transporting phosphotransferase system is dephosphorylated in the presence of glucose, and dephosphorylated HPr stimulates pyruvate kinase A in *V. vulnificus*. The increased pyruvate production then enables this bacterium to cope with H_2_O_2_ stress imposed by their fungal neighbors or other environmental factors.

Most bacteria have anti-oxidant defense systems to deal with oxidative stress by synthesizing catalase, which decomposes H_2_O_2_ into water and oxygen (Loewen, 1997). While *E. coli* possesses periplasmic catalase HPI (KatG) and the cytoplasmic catalase HPII (KatE) (Switala et al., 1990), *V. vulnificus* has been reported to express only the *katG* gene (Park et al., 2004). In the previous study, Park et al. (2004) reported that *V. vulnificus* cells were generally more sensitive to H_2_O_2_ than other enteric bacteria and also suggested that *V. vulnificus* may have mechanisms for oxidative stress response that are distinct from those found in *E. coli* (Park et al., 2004). For this reason, we speculated that *V. vulnificus* could use pyruvate as a defense mechanism to H_2_O_2_. Pyruvate is known to react with H_2_O_2_ and decompose it into CO_2_ and acetate to protect the cell from oxidative stress (Nath et al., 1994; Desagher et al., 1997; Giandomenico et al., 1997; Miwa et al., 2000). Therefore, *V. vulnificus* may increase pyruvate production via the HPr-PykA interaction to protect themselves from H_2_O_2_ stress caused by their fungal neighbors in the presence of glucose (**Figure 6**). The conversion of PEP to pyruvate is catalyzed by two pyruvate kinases, PykF and PykA. PykF, whose activity is not regulated by HPr, is essential for normal growth of *V. vulnificus*. PykA was dispensable under normal growth conditions in *V. vulnificus* (**Figure 5** and Supplementary Figures S1, S9A), suggesting that the protective effect of pyruvate is not due to an increased metabolic flux. The stimulation of PykA activity by dephosphorylated HPr results in the additional production of pyruvate by converting an incoming glucose.

The critical role of pyruvate in protection from H_2_O_2_ damages was also reported in other bacteria. Pyruvate protects pathogenic spirochetes, *Borrelia burgdorferi* and *Leptospira interrogans* from H_2_O_2_ toxicity (Troxell et al., 2014). In particular, *B. burgdorferi* lacks genes encoding catalase and therefore is sensitive to a micromolar dose of H_2_O_2_ generated by GOX. However, exogenously supplied pyruvate fully protected *B. burgdorferi* against H_2_O_2_ killing. Under the H_2_O_2_-challenged environment, *Pseudomonas fluorescens* also increases pyruvate production, although the precise mechanism is still unclear (Bignucolo et al., 2013). In this study, we uncovered a sophisticated strategy which allows *V. vulnificus* to cope with the killing action of competitors. This strategy, involving the stimulation of pyruvate kinase in the presence of glucose to cope with H_2_O_2_ stress, could be widespread in the aquatic environments, whereas H_2_O_2_ can be generated from both biotic and photochemical reactions (Baltar et al., 2013).

## Author Contributions

H-MK, C-KY, Y-HP, and Y-JS designed the study. H-MK, C-KY, and Y-HP performed the experiments. H-MK, C-KY, H-IH, Y-HP, and Y-JS analyzed the data and wrote the paper.

## Conflict of Interest Statement

The authors declare that the research was conducted in the absence of any commercial or financial relationships that could be construed as a potential conflict of interest.
