# Supplementary material for: Stimulation of Vibrio vulnificus Pyruvate Kinase in the Presence of Glucose to Cope With H2O2 Stress Generated by Its Competitors
Source: Front Microbiol. 2018 May 29;9:1112. doi: 10.3389/fmicb.2018.01112 (PMC5987630; doi:10.3389/fmicb.2018.01112)
Supplement: Supplementary file 1 [file Image_1.pdf]

Supplementary Material

Stimulation of *Vibrio vulnificus* Pyruvate Kinase in the Presence of Glucose to Cope with H_2O_2 Stress Generated by its Competitors

Hey-Min Kim[†‡], Chang-Kyu Yoon[†], Hyeong-In Ham, Yeong-Jae Seok[*] and Young-Ha Park[*]

School of Biological Sciences and Institute of Microbiology, Seoul National University, Seoul, Korea

***Correspondence:**
Yeong-Jae Seok
yjseok@snu.ac.kr
Young-Ha Park
winningyoung@gmail.com

[†]These authors contributed equally to this work

[‡]Present address: Department of Oral Biology, College of Dentistry, University of Florida, Gainesville, Florida, USA

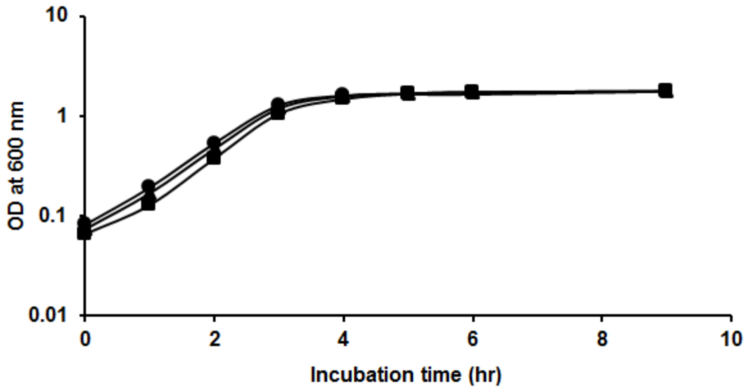

Supplementary Figure 1. Growth curves of wild-type *Vibrio vulnificus* CMCP6 (squares), the *pykA* mutant (triangles) and the complemented strain (circles) in LB medium supplemented with 2.5% NaCl (LBS medium).

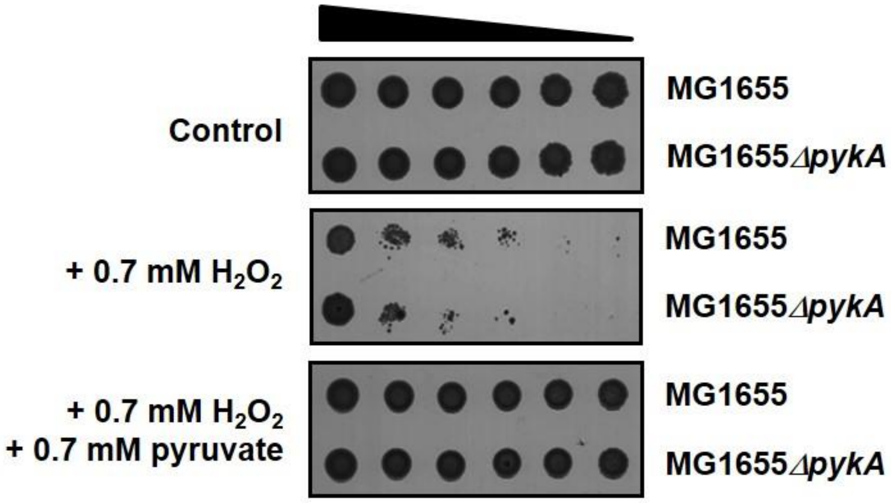

Supplementary Figure 2. Effect of *pykA* deletion on H_2O_2 stress resistance in *E. coli*. Stationary-phase cells of the indicated *E. coli* strains were serially diluted four-fold from 10^8 cells/ml and 2-μl aliquots were spotted onto LB medium containing different combinations of H_2O_2 and sodium pyruvate as indicated. Representative data from three independent experiments are shown.

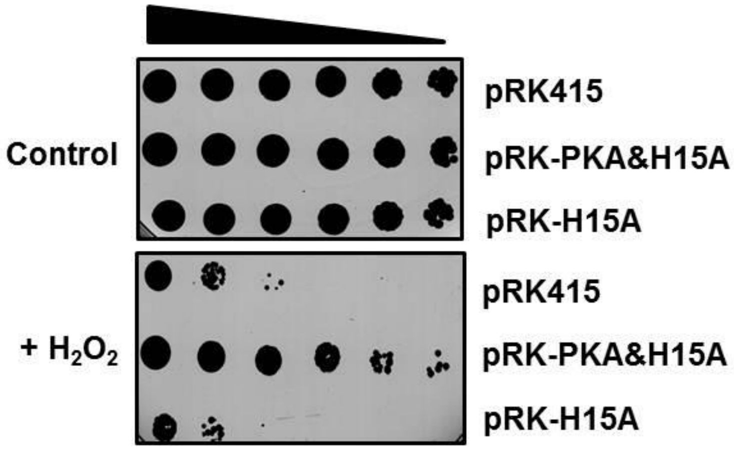

Supplementary Figure 3. Effect of dephospho-HPr on H_2O_2 resistance. The *pykA* mutant cells harboring indicated plasmids were serially diluted four-fold from 10^8 cells/ml, and 2-μl aliquots were spotted onto LBS medium in the absence or presence of H_2O_2. The pRK-PKA&H15A expresses both PykA and HPr(H15A) (a phosphorylation site mutant mimicking the dephosphorylated form) from their own promoters (Kim et al., 2015). Representative data from three independent experiments are shown.

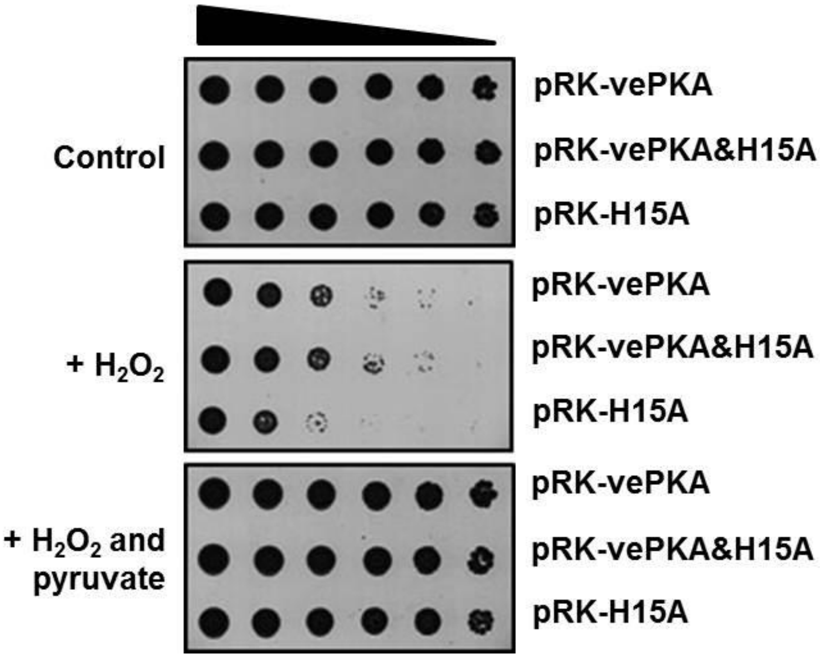

Supplementary Figure 4. Effects of chimeric PykA and dephospho-HPr on H_2O_2 resistance. The *pykA* mutant cells harboring the indicated plasmids were serially diluted four-fold from 10^8 cells/ml, and 2-μl aliquots were spotted onto LBS medium containing different combinations of H_2O_2 and sodium pyruvate as indicated. The pRK-vePKA expresses the chimeric protein made by fusing the N-terminal domain (amino acids 1-333) of *V. vulnificus* PykA to the C-terminal domain (amino acids 334-480) of *E. coli* PykA from the *V. vulnificus pykA* promoter and pRK-vePKA&H15A expresses both vePykA and HPr(H15A) from their own promoters(Kim et al., 2015). Representative data from three independent experiments are shown.

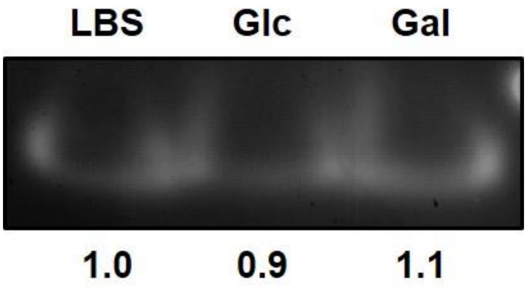

Supplementary Figure 5. The expression level of catalase in the presence of H_2O_2 in LBS medium containing glucose or galactose. *V. vulnificus* was cultured in LBS medium or LBS medium containing 0.2% glucose (Glc) or galactose (Gal) in the presence of 0.8 mM H_2O_2. When OD_{600} reached 0.8, the cells were collected by centrifugation and then disrupted. After separation on an 10% non-denaturing polyacrylamide gel, catalase activity was measured by staining the gel with a solution containing 2% $K_3Fe(CN)_6$ and 2% $FeCl_3$. Relative band intensities are indicated below the gel. Representative data from at least three independent experiments are shown.

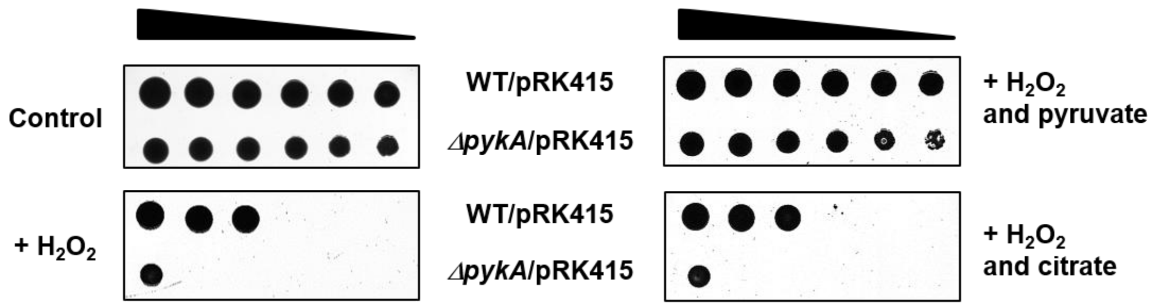

Supplementary Figure 6. Comparison of the effect of citrate with that of pyruvate on resistance to H_2O_2 stress. The indicated strains were serially diluted four-fold from 10^8 cells/ml, and 2-μl aliquots were spotted onto LBS medium containing indicated supplements. H_2O_2, pyruvate and citrate were added to 0.25 mM. Representative data from three independent experiments are shown.

A

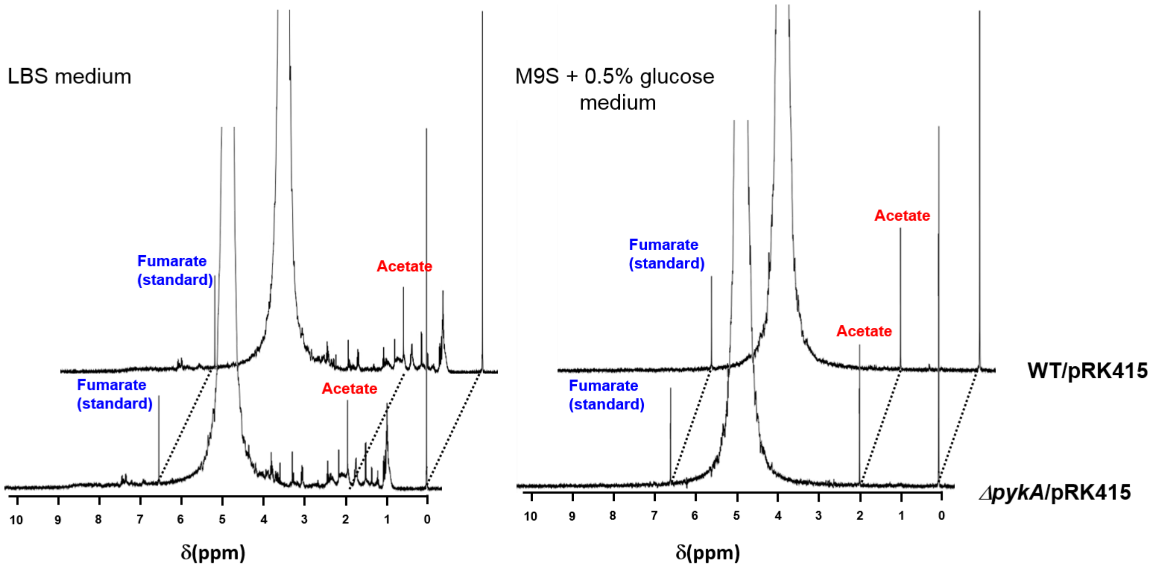

B

Strain	LBS			M9S + glucose		
	Exp. 1	Exp. 2	Exp. 3	Exp. 1	Exp. 2	Exp. 3
WT	6.61	6.75	3.87	3.23	5.60	6.21
Δ*pykA*	7.01	6.39	4.44	3.12	5.41	6.18

Supplementary Figure 7. Analysis of acetate concentrations by NMR Spectroscopy. **(A)** Wild-type CMCP6 and *pykA* mutant cells were grown in LBS or M9S medium containing 0.5% glucose. Aliquots of culture were taken at the late exponential growth phase and were centrifuged at 5,000 × *g* for 20 min at 4 °C. Supernatant solutions were used to analyze the extracellular metabolic products. The cell-free culture solutions (0.9 ml each) were mixed with D_2O (0.1 ml) and subjected to the NMR experiments. Fumarate (5 mM) was added to each sample as an internal control. The spectra were recorded at a ^{1}H resonance frequency of 400 MHz by use of a JeolJNM-LA400 spectrometer. Representative data from three independent experiments are shown. **(B)** Acetate concentrations in medium (mM). The peak area of acetate was compared with that of fumarate to analyze the concentration of acetate in medium in each of the three independent experiments.

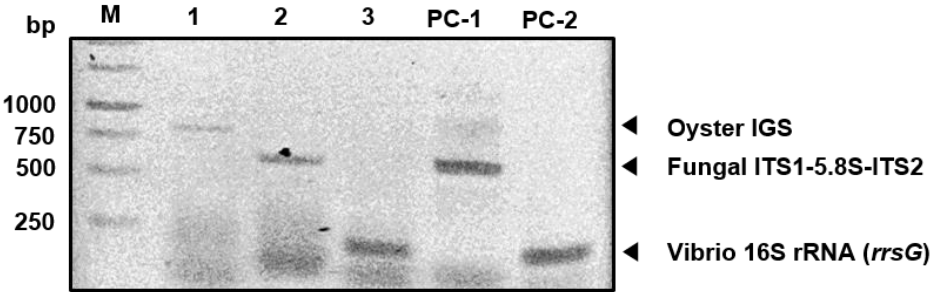

Supplementary Figure 8. Detection of fungi and bacteria from the digestive gland of raw oysters. Oysters (*Crassostrea gigas*) harvested from the west coast of Korea were aseptically opened and washed with 70% ethanol. Then whole DNA extracted from the digestive gland was used for PCRs with primers specific for the hypervariable mitochondrial DNA intergenic spacer (IGS) between $tRNA^{Cys}$-and $tRNA^{Asn}$ of *C. gigas* (lane 1, 826 bps) (Aranishi, 2006), the fungal ribosomal internal transcribed spacer ITS1-5.8S rDNA-ITS2 region (lane 2, ~600 bps) (Manter and Vivanco, 2007), and *Vibrio rrsG* encoding 16S rRNA (lane 3, 220 bps). The ITS 1-2 region was also amplified from genomic DNA of *Aspergillus* spp. as a positive control (lane PC-1), and *rrsG* from genomic DNA of *V. vulnificus* CMCP6 (PC-2). Primers used are: IGS-F**,** GCCCAAGAAATTGGCCTTTA; IGS-R**,** TGCCTTAAGCTTGGGCTACT; ITS-F, TCCGTAGGTGAACCTGCGG; ITS-R, TCCTCCGCTTATTGATATGC; rrsG-F, TTAGCCGGTGCTTCTTCTGT; and rrsG-R, CAGCCACACTGGAACTGAGA.

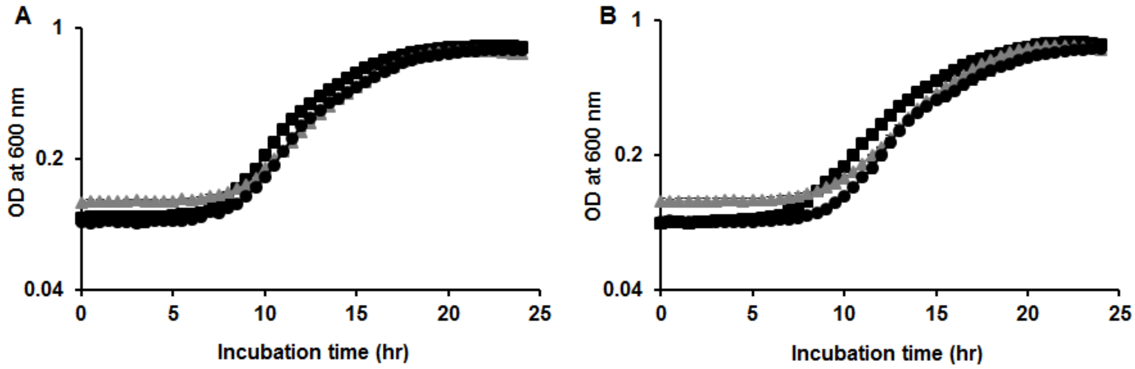

Supplementary Figure 9. *Aspergillus welwitschiae* culture filtrate in galactose medium has no growth inhibitory effect on *V. vulnificus* cells. **(A)** Wild-type *V. vulnificus* CMCP6 (squares), the *pykA* mutant (triangles) and the complemented strain (circles) were inoculated in M9 medium supplemented with 0.2% casamino acids and 2.5% NaCl (M9S medium) containing 0.2% galactose and growth was monitored by measuring the optical density at 600 nm. **(B)** *A. welwitschiae* was grown for 24 hours in M9S medium containing 0.2% galactose, and fungal cells were removed by filtration. The cell-free culture filtrate was then inoculated with *V. vulnificus* strains, and growth was monitored.

Supplementary references

Aranishi, F. (2006). A novel mitochondrial intergenic spacer reflecting population structure of Pacific oyster. *J Appl Genet* 47(2)**,** 119-123. doi: 10.1007/BF03194610.

Kim, H.M., Park, Y.H., Yoon, C.K., and Seok, Y.J. (2015). Histidine phosphocarrier protein regulates pyruvate kinase A activity in response to glucose in *Vibrio vulnificus*. *Mol Microbiol* 96(2)**,** 293-305. doi: 10.1111/mmi.12936.

Manter, D.K., and Vivanco, J.M. (2007). Use of the ITS primers, ITS1F and ITS4, to characterize fungal abundance and diversity in mixed-template samples by qPCR and length heterogeneity analysis. *J Microbiol Methods* 71(1)**,** 7-14. doi: 10.1016/j.mimet.2007.06.016.
